# Low-dose immunoglobulin G is not associated with mortality in patients with sepsis and septic shock

**DOI:** 10.1186/s13054-017-1764-4

**Published:** 2017-07-13

**Authors:** Yusuke Iizuka, Masamitsu Sanui, Yusuke Sasabuchi, Alan Kawarai Lefor, Mineji Hayakawa, Shinjiro Saito, Shigehiko Uchino, Kazuma Yamakawa, Daisuke Kudo, Kohei Takimoto, Toshihiko Mayumi, Takeo Azuhata, Fumihito Ito, Shodai Yoshihiro, Katsura Hayakawa, Tsuyoshi Nakashima, Takayuki Ogura, Eiichiro Noda, Yoshihiko Nakamura, Ryosuke Sekine, Yoshiaki Yoshikawa, Motohiro Sekino, Keiko Ueno, Yuko Okuda, Masayuki Watanabe, Akihito Tampo, Nobuyuki Saito, Yuya Kitai, Hiroki Takahashi, Iwao Kobayashi, Yutaka Kondo, Wataru Matsunaga, Sho Nachi, Toru Miike, Hiroshi Takahashi, Shuhei Takauji, Kensuke Umakoshi, Takafumi Todaka, Hiroshi Kodaira, Kohkichi Andoh, Takehiko Kasai, Yoshiaki Iwashita, Hideaki Arai, Masato Murata, Masahiro Yamane, Kazuhiro Shiga, Naoto Hori

**Affiliations:** 1 Department of Anesthesiology and Critical Care Medicine, Jichi Medical University Saitama Medical Center, 1-847 Amanuma, Omiya, Saitatama, Saitama 330-8503 Japan; 2 Department of Critical Care, Shonan Kamakura General Hospital, Kamakura, Japan; 3 Department of Clinical Epidemiology and Health Economics, School of Public Health, University of Tokyo, Tokyo, Japan; 4 Department of Surgery, Jichi Medical University, Tochigi, Japan; 5 Emergency and Critical Care Center, Hokkaido University Hospital, Sapporo, Japan; 6 Intensive Care Unit, Department of Anesthesiology, Jikei University School of Medicine, Tokyo, Japan; 7 Division of Trauma and Surgical Critical Care, Osaka General Medical Center, Osaka, Japan; 8 Division of Emergency and Critical Care Medicine, Tohoku University Graduate School of Medicine, Sendai, Japan; 9 Department of Anesthesiology and Intensive Care Medicine, Osaka University Graduate School of Medicine, Suita, Japan; 10 Department of Intensive Care Medicine, Kameda Medical Center, Kamogawa, Japan; 11 Department of Emergency Medicine, University of Occupational and Environmental Health, Kita-Kyushu, Japan; 12 Division of Emergency and Critical Care Medicine, Department of Acute Medicine, Nihon University School of Medicine, Tokyo, Japan; 13 Department of Emergency and Critical Care Medicine, Ohta General Hospital Foundation, Ohta Nishinouchi Hospital, Koriyama, Japan; 14 Pharmaceutical Department, JA Hiroshima General Hospital, Hiroshima, Japan; 15 Department of Emergency and Critical Care Medicine, Saitama Red Cross Hospital, Saitama, Japan; 16 Department of Emergency and Critical Care Medicine, Wakayama Medical University, Wakayama, Japan; 17 Department of Emergency Medicine and Critical Care Medicine, Advanced Medical Emergency Department and Critical Care Center, Japan Red Cross Maebashi Hospital, Maebashi, Japan; 18 Emergency and Critical Care Center, Kyushu University Hospital, Fukuoka, Japan; 19 Department of Emergency and Critical Care Medicine, Faculty of Medicine, Fukuoka University, Fukuoka, Japan; 20 Emergency Department, Ibaraki Prefectural Central Hospital, Kasama, Japan; 21 Division of Intensive Care, Nagasaki University Hospital, Nagasaki, Japan; 22 Department of Emergency and Critical Care Medicine, Tokyo Medical University, Hachioji Medical Center, Tokyo, Japan; 23 Department of Emergency and Critical Care Medicine, Kyoto Daiichi Red-Cross Hospital, Kyoto, Japan; 24 Intensive Care Unit, Saiseikai Yokohamasi Tobu Hospital, Yokohama, Japan; 25 Department of Emergency Medicine, Asahikawa Medical University, Asahikawa, Japan; 26 Shock and Trauma Center, Nippon Medical School Chiba Hokusoh Hospital, Inzai, Japan; 27 Emergency Medicine, Kameda Medical Center, Kamogawa, Japan; 28 Department of Traumatology and Acute Critical Medicine, Osaka University Graduate School of Medicine, Suita, Japan; 29 Emergency and Critical Care Medicine, Asahikawa Red Cross Hospital, Asahikawa, Japan; 30 Department of Emergency and Critical Care Medicine, Graduate School of Medicine, University of the Ryukyu, Nishihara, Japan; 31 Advanced Critical Care Center, Gifu University Hospital, Gifu, Japan; 32 Emergency and Critical Care Center, Saga University Hospital, Saga, Japan; 33 The Division of Cardiovascular Disease, Steel Memorial Muroran Hospital, Muroran, Japan; 34 Department of Emergency Medicine and Critical Care, Sapporo City General Hospital, Sapporo, Japan; 35 Division of Emergency Medicine, Ehime University Hospital, Toon, Japan; 36 Intensive Care Unit, Tomishiro Central Hospital, Tomishiro, Japan; 37 Department of Emergency Medicine, Akashi City Hospital, Akashi, Japan; 38 Department of Emergency and Critical Care, Sendai City Hospital, Sendai, Japan; 39 Emergency Department, Hakodate Municipal Hospital, Hakodate, Japan; 40 Emergency and Critical Care Center, Mie University Hospital, Tsu, Japan; 41 Department of Emergency Medicine, Gunma University, Maebashi, Japan; 42 Department of Anesthesia and Intensive Care, KKR Sapporo Medical Center, Sapporo, Japan; 43 Emergency and Critical Care Center, Seirei Mikatahara General Hospital, Hamamatsu, Japan; 44 Intensive Care Unit, Hyogo College of Medicine, Nishinomiya, Japan

**Keywords:** Polyclonal intravenous immunoglobulin G, IVIG, Propensity score, Sepsis, Infection, Adjunctive therapy

## Abstract

**Background:**

The administration of low-dose intravenous immunoglobulin G (IVIgG) (5 g/day for 3 days; approximate total 0.3 g/kg) is widely used as an adjunctive treatment for patients with sepsis in Japan, but its efficacy in the reduction of mortality has not been evaluated. We investigated whether the administration of low-dose IVIgG is associated with clinically important outcomes including intensive care unit (ICU) and in-hospital mortality.

**Methods:**

This is a post-hoc subgroup analysis of data from a retrospective cohort study, the Japan Septic Disseminated Intravascular Coagulation (JSEPTIC DIC) study. The JSEPTIC DIC study was conducted in 42 ICUs in 40 institutions throughout Japan, and it investigated associations between sepsis-related coagulopathy, anticoagulation therapies, and clinical outcomes of 3195 adult patients with sepsis and septic shock admitted to ICUs from January 2011 through December 2013. To investigate associations between low-dose IVIgG administration and mortalities, propensity score-based matching analysis was used.

**Results:**

IVIgG was administered to 960 patients (30.8%). Patients who received IVIgG were more severely ill than those who did not (Acute Physiology and Chronic Health Evaluation (APACHE) II score 24.2 ± 8.8 vs 22.6 ± 8.7, *p* < 0.001). They had higher ICU mortality (22.8% vs 17.4%, *p* < 0.001), but similar in-hospital mortality (34.4% vs 31.0%, *p* = 0.066). In propensity score-matched analysis, 653 pairs were created. Both ICU mortality and in-hospital mortality were similar between the two groups (21.0% vs 18.1%, *p* = 0.185, and 32.9% vs 28.6%, *p* = 0.093, respectively) using generalized estimating equations fitted with logistic regression models adjusted for other therapeutic interventions. The administration of IVIgG was not associated with ICU or in-hospital mortality (odds ratio (OR) 0.883; 95% confidence interval (CI) 0.655–1.192, *p* = 0.417, and OR 0.957, 95% CI, 0.724–1.265, *p* = 0.758, respectively).

**Conclusions:**

In this analysis of a large cohort of patients with sepsis and septic shock, the administration of low-dose IVIgG as an adjunctive therapy was not associated with a decrease in ICU or in-hospital mortality.

**Trial registration:**

University Hospital Medical Information Network Individual Clinical Trials Registry, UMIN-CTR000012543. Registered on 10 December 2013.

**Electronic supplementary material:**

The online version of this article (doi:10.1186/s13054-017-1764-4) contains supplementary material, which is available to authorized users.

## Background

To decrease the high mortality associated with sepsis [1], various adjunctive therapies have been suggested. The administration of low-dose intravenous immunoglobulin G (IVIgG) (5 g/day for 3 days, total 15 g) is widely used as an adjunctive therapy for patients with sepsis in Japan [2]. This practice was approved for clinical use based on a randomized controlled trial (RCT) by Masaoka et al. in 2000 [3] showing beneficial effects in septic patients. In this study, the administration of IVIgG, even at a low dose, was associated with earlier improvement of clinical signs and symptoms of sepsis. Although this study had a relatively large sample size (*n* = 682), it was not sufficiently powered for important outcomes including mortality, with a follow-up of only 7 days. Since that time, no high-quality studies have examined the efficacy of low-dose IVIgG in patients with sepsis. To investigate the association between the administration of low-dose IVIgG (5 g/day for 3 days) and clinically important outcomes in patients with sepsis (with or without septic shock), we reviewed a large Japanese database.

## Methods

This study is a post-hoc analysis of the database of the Japan Septic Disseminated Intravascular Coagulation (JSEPTIC DIC) study (University Hospital Medical Information Network Individual Clinical Trials Registry (UMIN-CTR000012543, http://www.umin.ac.jp/icdr/index-j.html). This study followed the principles of the Declaration of Helsinki and was approved by the Institutional Review Board of each participating hospital (Additional file 1: Table S1). Because of the anonymous and retrospective nature of this study, the board of each hospital waived the need for informed consent.

The JSEPTIC DIC study was conducted using data from 42 intensive care units (ICUs) in 40 institutions throughout Japan [4]. We reviewed all patients admitted to ICUs between January 2011 and December 2013 for the treatment of sepsis (formerly defined as severe sepsis by the International Sepsis Definitions Conference criteria, 2003 [5]). Patients younger than 16 years old and patients who developed sepsis after their ICU admission were excluded.

The following data were collected: ICU characteristics (number of beds, ICU model, preference for disseminated intravascular coagulation (DIC) therapy), age, gender, weight, admission route to the ICU, Acute Physiology and Chronic Health Evaluation (APACHE) II score, pre-existing organ dysfunction (using chronic health evaluation score in APACHE II), pre-existing hemostatic disorders, Sequential Organ Failure Assessment (SOFA) score (days 1, 3, and 7), systemic inflammatory response syndrome (SIRS) score (days 1, 3, and 7), primary infection site, blood culture results, microorganisms responsible for sepsis, daily results from laboratory tests during the first week after ICU admission, serum lactate levels (days 1, 3, and 7), administration of adjunctive medications (including anti-DIC drugs, other anticoagulants, IVIgG, and low-dose steroids) during the first week after ICU admission, transfusion volume (red blood cell (RBC) concentration, fresh frozen plasma (FFP), platelet concentrate) and bleeding complications during the first week after ICU admission, therapeutic interventions including surgical interventions at the infection site, renal replacement therapy, renal replacement therapy for non-renal indications, polymyxin B direct hemoperfusion, plasma exchange, extracorporeal membrane oxygenation (ECMO), and intra-aortic balloon pump use during the first week after ICU admission, duration of mechanical ventilation, vasoactive drugs and renal replacement therapy use up to 28 days after ICU admission, and ICU mortality and in-hospital mortality.

### Statistical analysis

Data are expressed as number (%), or median (interquartile range (IQR)), as appropriate. Patients who received IVIgG were compared with patients who did not receive IVIgG. To estimate the association between IVIgG therapy and mortality rates (ICU mortality and in-hospital mortality), multivariable logistic regression modeling and propensity score matching were used. We performed 1:1 nearest neighbor matching without replacement between the IVIgG and no-IVIgG groups based on estimated propensity scores for each patient. For propensity score matching, a caliper was set at 20% of the standard deviation of the logit of the propensity score. To calculate a propensity score, we fitted a logistic regression model for IVIgG administration adjusted for the following factors: ICU characteristics, age, gender, weight, admission route to the ICU, pre-existing organ dysfunction, pre-existing hemostatic disorders, APACHE II score, SOFA score of each organ on day 1, SIRS score on day 1, primary infection site, blood culture results (positive, negative, or not taken), causative microorganisms, surgical interventions to the infection source, and laboratory test results (white blood cell count, platelet count, hemoglobin level) on day 1. Other laboratory data collected (including fibrinogen, fibrin/fibrinogen degradation products, d-dimer, anti-thrombin, and lactate) were not used to estimate the propensity score since the proportion of missing data was >10%. Other therapeutic interventions were not included for the estimation of the propensity score because timing data of those interventions were not recorded in the database. Standardized difference was used to evaluate covariate balance, and an absolute standardized difference of >10% represents meaningful imbalance. To make the results more robust, we used generalized estimating equations fitted with logistic regression models in the matched groups to assess the association between IVIgG and mortality adjusting for clustering within hospitals and other therapeutic interventions which were not used to estimate the propensity score (anti-thrombin, recombinant human thrombomodulin, heparinoid, protease inhibitor, low-dose steroid, renal replacement therapy, renal replacement therapy for non-renal indications, polymyxin B direct hemoperfusion, plasma exchange, veno-arterial ECMO, veno-venous ECMO, intra-aortic balloon pump, and the volume of transfusion (RBC, FFP, platelet concentrate)).

The database does not include the exact timing of administration of IVIgG within the first week. The timing of administration in some patients might be better correlated with severity on day 2 or later. To adjust the severity within the first week, we added a supplemental analysis, using generalized estimating equations fitted with logistic regression models adjusting for clustering within hospitals, other therapeutic interventions, and SOFA score (each organ score) on days 3 and 7.

The survival curve was generated by the Kaplan-Meier method and hazard ratios for administration of IVIgG were estimated using the multivariable Cox regression model. Univariate differences between groups were assessed using the Mann-Whitney *U* test for continuous variables and chi-square test or Fisher’s exact test for categorical variables.

Interaction between high (29 and over, highest interquartile range) and low (less than 29) APACHE II score groups was tested using the Breslow-Day statistic in matched groups created by propensity score. Interactions between immunodeficiency and effects of IVIgG were evaluated by subgroups with and without immunodeficiency in the same way. A *p* value of 0.05 was considered statistically significant. All analyses were performed using IBM SPSS Statistics version 22 (IBM Corp., Armonk, NY, USA).

## Results

This database included 3195 patients, of which 3118 patients with no missing data on day 1 were enrolled. Propensity scores were estimated from these patients. Table 1 shows the characteristics of ICUs and patients in this study. IVIgG was administered to 960 patients (30.8%). IVIgG was used more often in larger ICUs, with an intensivist co-management model, and institutional preference to administer active DIC treatment. Patients who received IVIgG were younger, more severely ill (higher APACHE II, SOFA scores) and had lower platelet counts than those who did not. IVIgG was used more often in patients who were immunocompromised, had intra-abdominal infections, infection with gram-positive cocci, and needed surgical/nonsurgical drainage. After propensity score matching between the IVIgG and control groups, 653 pairs were obtained and baseline characteristics were well balanced between the groups (Table 1).

**Table 1:** Characteristics of ICUs and patients before and after propensity score matching

	Before propensity score matching			After propensity score matching		
	IVIgG(+)	IVIgG(–)	SD (%)	IVIgG(+)	IVIgG(–)	SD (%)
*n* (%)	960 (30.8)	2158 (69.2)		653	653	
ICU characteristics						
ICU beds volume	12 (10–19)	12 (8–19)	13.3	10 (8–16)	12 (8–18)	9.8
ICU type						
ER ICU	464 (48.3)	1077 (49.9)	3.2	308 (47.2)	308 (47.2)	0.0
General ICU	496 (51.7)	1081 (50.1)	3.2	345 (52.8)	345 (52.8)	0.0
Intensity of intensivists						
Closed ICU model	453 (47.2)	1102 (51.1)	7.8	329 (50.4)	326 (49.9)	1.0
Open ICU model	326 (34.0)	752 (34.8)	1.7	198 (30.3)	208 (31.9)	3.4
Intensivist co-management model	181 (18.9)	304 (14.1)	12.9	126 (19.3)	119 (18.2)	2.8
Preference to DIC therapy (%)						
Actively (radical)	763 (79.5)	1031 (47.8)	69.8	468 (71.7)	461 (70.6)	2.4
Not actively (conservative)	23 (4.4)	505 (23.4)	57.1	23 (3.5)	24 (3.7)	1.1
Neither (treat DIC occasionally)	174 (18.1)	622 (28.8)	25.5	162 (24.8)	168 (25.7)	2.1
Patient characteristics						
Age (years)	70 (61–79)	73 (63–81)	16.5	72 (62–80)	72 (62–80)	1.6
Gender (male) (%)	563 (58.6)	1307 (60.6)	4.1	395 (60.5)	394 (60.3)	0.4
Weight (kg)	55.9 (47.2–65.0)	54.0 (46.2–64.0)	5.8	55.7 (47.0–65.0)	54.0 (46.0–63.4)	7.2
Prior location (%)						
Outpatient (ER)	354 (36.9)	1049 (48.6)	23.8	272 (41.7)	287 (44.0)	4.6
In-hospital (general ward)	329 (34.3)	588 (27.2)	15.4	189 (28.9)	184 (28.2)	4.6
Transferred from other hospital	277 (28.9)	521 (24.1)	10.9	192 (29.4)	182 (27.9)	3.3
SIRS score day1	3 (2–4)	3 (2–4)	4.4	3 (2–4)	3 (2–4)	1.1
APACHEII	24 (17–30)	22 (16–28)	18.3	23 (17–30)	23 (17–29)	5.4
SOFA score day1						
Respiratory	2 (1–3)	2 (1–3)	11.6	2 (1–3)	2 (1–3)	3.3
Hematologic	1 (0–2)	1 (0–2)	24.4	1 (0–2)	1 (0–2)	0.8
Hepatic	0 (0–1)	0 (0–1)	12.1	0 (0–1)	0 (0–1)	0.0
Cardiovascular	3 (2–4)	3 (1–4)	29.6	3 (1–4)	3 (1–4)	5.8
Neurologic	1 (0–3)	1 (0–3)	9.9	1 (0–3)	1 (0–3)	4.9
Renal	2 (0–3)	1 (0–3)	15.1	2 (0–3)	2 (0–3)	7.6
Laboratory data day1						
White blood cell count (10^9^/l)	10.6 (3.3–17.9)	11.5 (5.5–17.7)	1.8	11.8 (3.8–18.8)	11.4 (5.1–17.7)	2.3
Hemoglobin (g/dl)	10.5 (8.9–12.2)	10.7 (9.0–12.6)	6.7	10.6 (9.0–12.3)	10.6 (9.1–12.6)	0.8
Platelet count (10^9^/l)	107 (54–172)	130 (72–203)	21.1	118 (64–188)	125 (66–194)	1.6
Comorbidity						
Liver	52 (5.4)	75 (3.5)	9.2	25 (3.8)	34 (5.2)	6.8
Respiratory	38 (4.0)	82 (3.8)	1.0	29 (4.4)	29 (4.4)	0.0
Cardiovascular	65 (6.8)	113 (5.2)	6.7	40 (6.1)	45 (6.9)	3.2
Renal	82 (8.5)	173 (8.0)	1.8	50 (7.7)	57 (8.7)	3.6
Immunocompromised	174 (18.1)	310 (14.4)	10.0	107 (16.4)	99 (15.2)	3.3
Pre-existing coagulopathy (%)						
Cirrhosis/liver failure	45 (4.7)	75 (3.5)	6.1	24 (3.7)	28 (4.3)	3.1
Chemotherapy	48 (5.0)	94 (4.4)	2.8	31 (4.7)	35 (5.4)	3.2
Hematologic malignancy	40 (4.2)	58 (2.7)	8.2	16 (2.5)	24 (3.7)	6.9
Medication of warfarin	31 (3.2)	120 (5.6)	11.7	29 (4.4)	26 (4.0)	2.0
Others	19 (2.0)	36 (1.7)	2.2	16 (2.5)	14 (2.1)	2.7
Infection site (%)						
Abdomen	346 (36.0)	674 (31.2)	10.2	234 (35.8)	222 (34.0)	3.8
Lung	226 (23.5)	571 (26.5)	6.9	159 (24.3)	162 (24.8)	1.2
Urinary tract	133 (13.9)	361 (16.7)	7.8	98 (15.0)	102 (15.6)	1.7
Musculoskeletal	128 (13.3)	238 (11.0)	7.0	75 (11.5)	75 (11.5)	0.0
Infectious endocarditis	21 (2.2)	47 (2.0)	1.4	14 (2.1)	12 (1.2)	7.1
Others	14 (1.5)	44 (2.0)	3.8	13 (2.0)	16 (2.5)	3.4
Central nerve system	19 (2.0)	42 (1.9)	0.7	14 (2.1)	14 (2.1)	0.0
CRBSI	21 (2.2)	23 (1.1)	8.6	7 (1.1)	9 (1.4)	2.6
Unknown	52 (5.4)	162 (7.5)	8.5	39 (6.0)	41 (6.3)	1.3
Blood culture (%)						
Positive	438 (45.6)	931 (43.1)	5.0	286 (43.8)	282 (43.2)	1.2
Negative	464 (48.3)	1100 (51.0)	5.4	324 (49.6)	320 (49.0)	1.2
Not taken	58 (6.0)	127 (5.9)	0.4	43 (6.6)	51 (7.8)	4.6
Microorganism (%)						
Gram-negative rod	379 (39.5)	753 (34.9)	9.5	254 (38.9)	240 (36.8)	4.3
Gram-positive cocci	268 (27.9)	458 (21.2)	15.6	150 (23.0)	165 (25.3)	5.4
Combined	110 (11.5)	287 (13.3)	5.5	86 (13.2)	81 (12.4)	2.4
Others	13 (1.4)	45 (2.1)	5.3	10 (1.5)	9 (1.4)	0.8
Fungus	13 (1.4)	42 (1.9)	3.9	11 (1.7)	8 (1.2)	4.2
Virus	5 (0.5)	23 (1.1)	6.7	5 (0.8)	4 (0.6)	2.4
Unknown	172 (17.9)	550 (25.5)	18.5	137 (21.0)	146 (22.4)	3.4
Surgical intervention/drainage (%)	466 (48.5)	848 (39.3)	18.6	353 (54.1)	351 (53.8)	0.6

Values are shown as *n* (%) or median (interquartile range) as appropriate

*APACHE* Acute Physiology and Chronic Health Evaluation, *CRBSI* catheter-related blood stream infection, *DIC* disseminated intravascular coagulation, *ER* emergency room, *ICU* intensive care unit, *IVIgG* intravenous immunoglobulin G, *SD* standardized difference, *SIRS* systemic inflammatory response syndrome, *SOFA* Sequential Organ Failure Assessment

The proportion of patients receiving adjunctive interventions was compared between the groups treated with and without IVIgG (Table 2). Patients given IVIgG required larger volumes of transfusion and also used more adjunctive therapies including anti-DIC medications (antithrombin, recombinant human soluble thrombomodulin, heparinoid, protease inhibitor), low-dose steroids, renal replacement therapy for non-renal indications, polymyxin B direct hemoperfusion, and plasma exchange before propensity score matching. The proportion of patients receiving heparinoid and plasma exchange did not differ between the groups after propensity score matching; however, other adjunctive interventions and transfusions were used more in the IVIgG group.

**Table 2:** Other adjunctive treatments used in patients treated with or without IVIgG

	Before propensity score matching			After propensity score matching		
	IVIgG(+) (*n* = 960)	IVIgG(–) (*n* = 2158)	*p* value	IVIgG(+) (*n* = 653)	IVIgG(–) (*n* = 653)	*p* value
Adjunctive treatments of sepsis (%)						
Anti-DIC therapy						
Antithrombin	530 (55.2)	439 (20.3)	<0.001	337 (51.6)	188 (28.8)	<0.001
Recombinant human soluble thrombomodulin	431 (50.7)	419 (19.4)	<0.001	270 (41.3)	170 (26.0)	<0.001
Heparinoid	66 (6.9)	93 (4.3)	0.002	44 (6.7)	35 (5.4)	0.296
Protease inhibitor	195 (20.3)	192 (8.9)	<0.001	129 (19.8)	92 (14.1)	0.006
Low-dose steroid	364 (37.9)	399 (18.5)	<0.001	242 (37.1)	147 (22.5)	<0.001
Renal replacement therapy	377 (39.3)	501 (23.2)	<0.001	234 (35.8)	165 (25.3)	<0.001
Renal replacement therapy for non-renal indications	135 (14.1)	129 (6.0)	<0.001	88 (13.5)	63 (9.6)	0.031
Polymyxin B direct hemoperfusion	327 (34.1)	357 (16.5)	<0.001	207 (31.7)	142 (21.7)	<0.001
Plasma exchange	17 (1.8)	12 (0.6)	0.002	11 (1.7)	5 (0.8)	0.131
Veno-arterial ECMO	7 (0.7)	21 (1.0)	0.330	6 (0.9)	4 (0.6)	0.525
Veno-venous ECMO	14 (1.5)	26 (1.2)	0.335	7 (1.1)	5 (0.8)	0.562
Intra-aortic balloon pumping	5 (0.5)	8 (0.4)	0.369	3 (0.5)	0 (0.0)	0.083
Transfusion (units)						
RBC concentration	2 (0–6)	0 (0–4)	<0.001	2 (0–6)	0 (0–4)	<0.001
FFP	0 (0–10)	0 (0–0)	<0.001	0 (0–10)	0 (0–4)	<0.001
Platelet concentration	0 (0–20)	0 (0–0)	<0.001	0 (0–10)	0 (0–0)	0.002

*DIC* disseminated intravascular coagulation, *FFP* fresh frozen plasma, *ECMO* extracorporeal membrane oxygenation, *RBC* red blood cell

Before propensity score matching, ICU mortality was significantly higher in the IVIgG group (22.8% vs 17.4%, *p* < 0.001), and in-hospital mortality was higher in the IVIgG group, but did not differ statistically (34.4% vs 31.0%, *p* = 0.066). After propensity score matching, ICU mortality and in-hospital mortality (21.0% vs 18.1%, *p* = 0.185, and 32.9% vs 28.6%, *p* = 0.093, respectively) were not significantly different between the groups (Table 3). The duration of mechanical ventilation, use of vasoactive drugs and renal replacement therapy, and length of ICU stay were longer in the IVIgG group, but the length of hospital stay was similar between the groups before and after propensity score matching. To assess the association of other therapeutic interventions and mortality, we used generalized estimating equations fitted with logistic regression models in the propensity score-matched groups. In this adjusted model, IVIgG was not associated with a decrease in either ICU or in-hospital mortality (odds ratio (OR) 0.883, 95% confidence interval (CI) 0.655–1.192, *p* = 0.417, and OR 0.957, 95% CI 0.724–1.265, *p* = 0.758, respectively) (Table 4). Supplemental analyses (generalized estimating equations fitted with logistic regression models adjusting for clustering within hospitals, other therapeutic interventions, and SOFA score on days 3 and 7) also showed that IVIgG was not associated with ICU mortality or in-hospital mortality (Additional file 1: Tables S2 and S3).

**Table 3:** Mortality and duration of major therapeutic interventions used, and length of stay in ICU and hospital for patients treated with or without IVIgG

	Before propensity score matching			After propensity score matching		
	IVIgG(+) (*n* = 960)	IVIgG(–) (*n* = 2158)	*p* value	IVIgG(+) (*n* = 653)	IVIgG(–) (*n* = 653)	*p* value
Mortality						
ICU mortality (%)	219 (22.8)	376 (17.4)	<0.001	137 (21.0)	118 (18.1)	0.185
In-hospital mortality (%)	330 (34.4)	670 (31.0)	0.066	215 (32.9)	187 (28.6)	0.093
Duration of major intervention uses up to 28 days and length of stay (days)						
Duration of mechanical ventilation	5 (2–12)	3 (0–9)	<0.001	5 (2–12)	3 (0–9)	<0.001
Duration of vasoactive drugs	3 (2–7)	3 (0–5)	<0.001	3 (2–6.5)	3 (1–6)	0.004
Duration of renal replacement therapy	1 (0–5)	0 (0–2)	<0.001	0 (0–4)	0 (0–2)	<0.001
Length of stay in ICU	8 (4–15)	6 (3–13)	<0.001	8 (4–15)	7 (4–13)	0.012
Length of stay from ICU admission to discharge	28 (14–55)	27 (13–53)	0.310	28 (14–50)	27 (13–52.5)	0.743

*ICU* intensive care unit, *IVIgG* intravenous immunoglobulin G

**Table 4:** ICU and in-hospital mortality rates in unadjusted and adjusted model

	IVIgG(+)	IVIgG(–)	Odds ratio (95% CI)	*p* value
ICU mortality (%)				
Unadjusted	137/653 (21.0)	118/653 (18.1)	1.204 (0.915–1.584)	0.185
Adjusted			0.883 (0.655–1.192)	0.417
In-hospital mortality (%)				
Unadjusted	215/653 (32.9)	187/653 (28.6)	1.223 (0.967–1.548)	0.093
Adjusted			0.957 (0.724–1.265)	0.758

*CI* confidence Interval, *ICU* intensive care unit, *IVIgG* intravenous immunoglobulin G

**Fig. 1:**
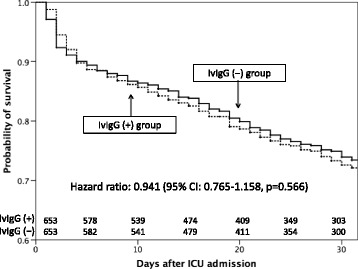
Kaplan-Meier survival curves for propensity score-matched groups with and without IVIgG treatment. *CI* confidence interval, *ICU* intensive care unit, *IVIgG* intravenous immunoglobulin G

Figure 1 shows Kaplan-Meier survival curves, and Cox regression analysis revealed no significant difference in the in-hospital survival after ICU admission between the propensity score-matched groups (hazard ratio (HR) 0.941, 95% CI 0.765–1.158, *p* = 0.566).

Interactions between high APACHE II score (29 and over, highest interquartile range in propensity score-matched groups) and IVIgG administration, and interactions between immunodeficiency and IVIgG use and mortality were also evaluated. There were no interactions between high APACHE II score and IVIgG use on ICU mortality (*p* value for interaction = 0.095), and in-hospital mortality (*p* value for interaction = 0.218). There was no interaction between immunodeficiency and IVIgG use on ICU mortality (*p* value for interaction = 0.247), or in-hospital mortality (*p* value for interaction = 0.378).

## Discussion

This is the first large cohort study to evaluate the association between low-dose IVIgG and clinically important outcomes in patients with sepsis and septic shock in Japan. Propensity-matched analysis shows that low-dose IVIgG administration (total 15 g: approximate total 0.3 g/kg) is not associated with a decrease in either ICU mortality or in-hospital mortality. Interactions between high APACHE II score and between immunodeficiency and IVIgG use were not detected with mortality.

Recently, Tagami et al. [6] reported similar results from a large nationwide database (*n* = 8264) showing that the use of low-dose IVIgG does not reduce mortality in patients with septic shock and pneumonia undergoing mechanical ventilation. They also reported a lack of effect of low-dose IVIgG in patients undergoing mechanical ventilation with septic shock who underwent laparotomy for lower gastrointestinal perforations [7]. These studies used the database of the Japanese Diagnosis Procedure Combination which includes administrative claims and discharge abstract data, but does not include severity scores such as the APACHE II or SOFA scores. The database used in the present study includes more clinically relevant indices which potentially reflect outcomes. The large database used by Tagami et al. and the more clinically relevant database used in this study both support the recommendation against the use of IVIgG in the Surviving Sepsis Campaign 2016 [8].

Several reasons can be postulated why the present study failed to show a benefit of low-dose IVIgG administration. First, IVIgG may not reduce mortality, although IVIgG has several theoretical advantages in the treatment of patients with sepsis. The mechanisms of these advantages are multifaceted, including pathogen recognition, clearance, and toxin scavenging. IVIgG preparations may have beneficial effects on the host response to infection [9, 10]. However, the current consensus does not favor the use of IVIgG [8].

There were two RCTs with a relatively large sample size (>500 patients) to evaluate the efficacy of IVIgG in the treatment of patients with sepsis. The SBITS study was conducted in Germany, and showed that the administration of 0.9 g/kg IVIgG (0.6 g/kg day 1; day 2, 0.3 g/kg; total 0.9 g/kg) did not decrease the 28-day mortality in patients with severe sepsis [11]. This study also reported a shortened duration of mechanical ventilation in the IVIgG group. The SBITS study was well designed with a large sample size (*n* = 653), but failed to show beneficial effects of IVIgG on mortality. Another large RCT (*n* = 682) was performed in Japan in 2000 by Masaoka et al. [3] and reported that the administration of low-dose IVIgG (5 g/day for 3 days) to patients with sepsis (most patients included were immunocompromised with hematologic diseases) resulted in earlier improvement of clinical parameters and recovery. The study had a large sample size, but many limitations. There was no placebo group, and intention-to-treat analysis was not used. The effects of IVIgG on survival were not examined in that study. A Cochrane database of systematic reviews showed that the administration of IVIgG is associated with a significant reduction in mortality in patients with sepsis compared with placebo or no intervention (relative risk 0.81, 95% CI 0.70–0.93), but sensitivity analysis of trials with a low risk of bias showed no reduction in mortality with IVIgG in adults (relative risk 0.97, 95% CI 0.81–1.15; five trials, *n* = 945) [12]. These clinical data did not suggest a beneficial effect of IVIgG on mortality in patients with sepsis.

Second, the dose of IVIgG given may be insufficient in patients with sepsis. Turgeon et al. [13] reported in a meta-analysis that regimens using 1 g or more per kilogram, duration of therapy longer than 2 days, and use of IVIgG in more severely ill patients is associated with increased survival [13]. The dose of IVIgG given in the present study is approximately 0.3 g/kg, which is the lowest among the 20 RCTs used in the meta-analysis.

This study has several acknowledged limitations. Data regarding the administration of IVIgG during the first week after ICU admission is limited to a binary condition (yes or no). The exact timing, dose, and duration of administration of IVIgG was not available in the database. The timing of administration in some patients might be better correlated with severity on day 2 or later. To adjust the severity within the first week, we added supplemental analyses and the results were not changed. We could not evaluate the effect of IVIgG administered after the first week. Some institutions may have administered larger or smaller doses of IVIgG than 5 g/day for 3 days. However, the average dose of IVIgG administered in this database may be close to 15 g, since no more than 5 g/day for 3 days is the dose approved (and reimbursed) by the Ministry of Health, Labor and Welfare in Japan. Second, the retrospective nature of this study may introduce residual confounding factors not accounted for by the propensity matching analysis. Although this database has a large number of clinically relevant factors potentially affecting the outcomes of patients with sepsis, residual confounding factors might bias the results. Third, the effects of other therapeutic interventions on mortality are unclear. In fact, more adjunctive therapeutic interventions were used in patients who received IVIgG (Table 2). In the propensity score analysis, we did not include those interventions because whether other interventions were administered before or after the administration of IVIgG is unknown. To compensate for this limitation, we added multivariable logistic regression analysis including adjunctive therapeutic interventions as independent variables, which resulted in negative results (Table 4). Although the possibility of reverse causality cannot be excluded, the effect of adjunctive interventions appears to be minimal. Fourth, interactions between high APACHE II score, immunodeficiency, and the effects of IVIgG on mortality cannot be totally excluded. In this context, we added an analysis of those interactions, with negative results.

## Conclusions

In this large cohort of patients with sepsis (with or without septic shock), the administration of low-dose IVIgG (approximate total 0.3 g/kg) as adjunctive therapy was not independently associated with ICU or in-hospital mortality. Based on the results of this study and previous ones, the clinical indications for the use of low-dose IVIgG in patients with sepsis cannot be recommended at this time.

## Additional file

Additional file 1: Table S1. Ethical approval information of each participating hospital. **Table S2** Factors associated with ICU mortality using generalized estimating equations fitted with logistic regression models adjusting for clustering within hospitals, other therapeutic interventions, and SOFA score on days 3 and 7. **Table S3** Factors associated with in-hospital mortality using generalized estimating equations fitted with logistic regression models adjusting for clustering within hospitals, other therapeutic interventions, and SOFA score on days 3 and 7. (DOCX 26 kb)

## Abbreviations

APACHE: Acute Physiology and Chronic Health Evaluation
CI: Confidence interval
DIC: Disseminated intravascular coagulation
ECMO: Extracorporeal membrane oxygenation
FFP: Fresh frozen plasma
ICU: Intensive care unit
IVIgG: Intravenous immunoglobulin G
JSEPTIC DIC: Japan Septic Disseminated Intravascular Coagulation
OR: Odds ratio
RBC: Red blood cell concentration
RCT: Randomized controlled trial
SIRS: Systemic inflammatory response syndrome
SOFA: Sequential Organ Failure Assessment
