# Supplementary material for: Low-dose immunoglobulin G is not associated with mortality in patients with sepsis and septic shock
Source: Crit Care. 2017 Jul 13;21:181. doi: 10.1186/s13054-017-1764-4 (PMC5508630; doi:10.1186/s13054-017-1764-4)
Supplement: Additional file 1: Table S1. — Ethical approval information of each participating hospital. Table S2 Factors associated with ICU mortality using generalized estimating equations fitted with logistic regression models adjusting for clustering within hospitals, other therapeutic interventions, and SOFA score on days 3 and 7. Table S3 Factors associated with in-hospital mortality using generalized estimating equations fitted with logistic regression models adjusting for clustering within hospitals, other therapeutic interventions, and SOFA score on days 3 and 7. (DOCX 26 kb) [file 13054_2017_1764_MOESM1_ESM.docx]

**Additional file 1**

**Table S1** Ethical approval information of each participating hospital

Jichi Medical University Saitama Medical Center (Bioethics Committee for Clinical Research, Saitama Medical Center, Jichi Medical University, #Rin13-98), Shonan Kamakura General Hospital (The Institutional Review Board of Shonan Kamakura General Hospital, #Syokama20140220-1), Hokkaido University Hospital (The Institutional Review Board of Hokkaido University Hospital, #Ji013-0246), Jikei University School of Medicine (The Ethics Committee of the Jikei University School of Medicine for Biomedical Research, #25-315 7450), Osaka General Medical Center (The Institutional Review Board of Osaka General Medical Center, #25-2050), Tohoku University Graduate School of Medicine (Institutional Review Board of Tohoku University School of Medicine, #2013-1-467), Osaka University Graduate School of Medicine (Ethical Review Board of Osaka University Hospital, #13465 and #13487), University of Occupational and Environmental Health (Ethics Committee of Medical Research, University of Occupational and Environmental Health, #H26-040), Nihon University School of Medicine (Research Review Board of Nihon University School of Medicine, Itabashi Hospital, #RK-140411-7), Ohta General Hospital Foundation Ohta Nishinouchi Hospital (The Institutional Review Board of Ohta Nishinouchi Hospital, no specific identification number), JA Hiroshima General Hospital (The Institutional Review Board of JA Hiroshima General Hospital, #14-6), Saitama Red Cross Hospital (Hospital Ethical Committee of Saitama Red Cross, #20140514-2), Wakayama Medical University (The Ethical Review Board of Wakayama Medical University, #1365), Japan Red Cross Maebashi Hospital (Research Review Board of Japan Red Cross Maebashi Hospital, #25-36), Kyushu University Hospital (Kyushu University Institutional Review Board for Clinical Research, #26-146), Fukuoka University Hospital (Institutional Review Board of Fukuoka University Hospital, #14-4-15), Ibaraki Prefectural Central Hospital (Clinical Research Ethics Review Committee of Ibaraki Prefectural Central Hospital, #25-79), Nagasaki University Hospital (the Institutional Review Board of Nagasaki University Hospital, #14012754), Tokyo Medical University Hachioji Medical Center (The Institutional Review Board of Tokyo Medical University Hachioji Medical Center, #H-13), Kyoto Daiichi Red Cross Hospital (The Ethical Committee of Kyoto Daiichi Red Cross Hospital, #362), Saiseikai Yokohama Eastern Hospital (Saiseikai Yokohama Eastern Hospital Ethics Committee, #2013065), Asahikawa Medical University (Asahikawa Medical University Research Ethics Committee, #1737), Nippon Medical School Chiba Hokusoh Hospital (The Ethical Review Board of Nippon Medical School Chiba Hokusoh Hospital, #409), Kameda Medical Center (Kameda Medical Center, Research Ethics Committee, #13-089), Asahikawa Red Cross Hospital (Clinical Research Ethics Review Committee of Asahikawa Redcross Hospital, #201336-2), Graduate School of Medicine, University of the Ryukyus (The Ethical Committee of the University of Ryukyus, #208), Gifu University Hospital (Medical Review Board of Gifu University Graduate School of Medicine, #26-30), Saga University Hospital (Ethics Committee , Faculty of Medicine , Saga University, #26-6), Steel Memorial Muroran Hospital (The Ethical Committee of Steel Memorial Murrain Hospital, no specific identification number), Sapporo City General Hospital (The Ethical Committee of Sapporo City General Hospital, #H25-047-191), Ehime University Hospital (the Institutional Review Board of Ehime University Hospital, #Aidaiibyorin1402011), Tomishiro Central Hospital (The Institutional Review Board of Tomishiro Central Hospital, #H26R002), Akashi City Hospital (The Institutional Review Board of Akashi City Hospital, # 2014-001), Sendai City Hospital (The Ethical Committee of Sendai city hospital, #Senbyoso595), Hakodate Municipal Hospital (Ethical Review Board of Hakodate Municipal Hospital, no specific identification number), Mie University Hospital (the Clinical Research Ethics Review Committee of Mie University Hospital, #2740), Gunma University (Institutional Review Board of Gunma University Hospital, no specific identification number), KKR Sapporo Medical Center (Ethical Review Board of KKR Sapporo Medical Center, #25-25), Seirei Mikatahara General Hospital (The Ethical Committee of Seirei Mikatahara General Hospital, #13-27), Hyogo College of Medicine (The Ethics Review Board of Hyogo College of Medicine, #1681)

**Table S2** Factors associated with ICU mortality using generalized estimating equations fitted with logistic regression models adjusting for clustering within hospitals, other therapeutic interventions and SOFA score on days 3 and 7.

|  | Odds ratio (95% CI) | p value |
| --- | --- | --- |
| Adjunctive treatment of sepsis |  |  |
| Anti-DIC therapy |  |  |
| Antithrombin | 1.644 (0.871-3.102) | 0.125 |
| Recombinant human soluble thrombomodulin | 0.779 (0.398-1.528) | 0.468 |
| Heparinoid | 1.829 (0.842-3.971) | 0.127 |
| Protease inhibitor | 0.725 (0.408-1.288) | 0.273 |
| **IVIgG** | 0.815 (0.456-1.457) | 0.49 |
| Low dose steroid | 1.341 (0.783-2.298) | 0.285 |
| Renal replacement therapy | 1.819 (0.852-3.886) | 0.122 |
| Renal replacement therapy for non-renal indications | 1.394 (0.636-3.053) | 0.407 |
| Polymyxin B direct hemoperfusion | 0.820 (0.459-1.466) | 0.503 |
| Plasma exchange | 1.261 (0.234-6.798) | 0.787 |
| Veno-arterial ECMO | 1.588 (0.002-1577) | 0.896 |
| Veno-venous ECMO | 0.954 (0.237-3.834) | 0.947 |
| Transfusion |  |  |
| RBC | 1.074 (1.030-1.119) | 0.001 |
| FFP | 0.996 (0.985-1.008) | 0.55 |
| Platelet concentration | 0.994 (0.986-1.003) | 0.183 |
| SOFA score day3 |  |  |
| Respiratory | 1.027 (0.834-1.264) | 0.803 |
| Hematologic | 0.776 (0.609-0.990) | 0.041 |
| Hepatic | 0.730 (0.521-1.022) | 0.067 |
| Cardiovascular | 0.901 (0.747-1.087) | 0.278 |
| Neurologic | 1.049 (0.704-1.563) | 0.816 |
| Renal | 0.954 (0.719-1.265) | 0.743 |
| SOFA score day7 |  |  |
| Respiratory | 1.810 (1.493-2.196) | <0.001 |
| Hematologic | 1.498 (1.112-2.018) | 0.008 |
| Hepatic | 1.397 (1.051-1.856) | 0.021 |
| Cardiovascular | 1.198 (0.964-1.489) | 0.103 |
| Neurologic | 1.378 (0.987-1.925) | 0.06 |
| Renal | 1.358 (1.027-1.796) | 0.032 |
| 181 patients were excluded from the analysis since SOFA score at day 3 or was not recorded. Since the number of patients was small, intra-aortic balloon pumping was not included in the logistic regression model.  Abbreviation: DIC; disseminated intravascular coagulation, ECMO; extracorporeal membrane oxygenation, RBC; red blood cell concentration, FFP; fresh frozen plasma, CI; confidence Interval | | |
|  |  |  |
|  |  |  |
|  |  |  |
|  |  |  |
|  |  |  |

**Table S3** Factors associated with in-hospital mortality using generalized estimating equations fitted with logistic regression models adjusting for clustering within hospitals, other therapeutic interventions and SOFA score on days 3 and 7.

|  | Odds ratio (95% CI) | p value |
| --- | --- | --- |
| Adjunctive treatment of sepsis |  |  |
| Anti-DIC therapy |  |  |
| Antithrombin | 1.135 (0.794-1.624) | 0.487 |
| Recombinant human soluble thrombomodulin | 0.656 (0.421-1.023) | 0.468 |
| Heparinoid | 0.898 (0.532-1.515) | 0.687 |
| Protease inhibitor | 0.948 (0.615-1.462) | 0.809 |
| **IVIgG** | 0.965 (0.668-1.394) | 0.849 |
| Low dose steroid | 1.491 (1.030-2.157) | 0.034 |
| Renal replacement therapy | 1.261 (0.753-2.111) | 0.378 |
| Renal replacement therapy for non-renal indications | 0.733 (0.417-1.288) | 0.28 |
| Polymyxin B direct hemoperfusion | 0.739 (0.539-1.014) | 0.061 |
| Plasma exchange | 2.037 (0.366-11.34) | 0.417 |
| Veno-arterial ECMO | 2.002 (0.053-75.96) | 0.14 |
| Veno-venous ECMO | 0.505 (0.110-2.317) | 0.38 |
| Transfusion |  |  |
| RBC | 1.037 (1.003-1.072) | 0.032 |
| FFP | 0.995 (0.985-1.006) | 0.373 |
| Platelet concentration | 1.000 (0.988-1.012) | 0.95 |
| SOFA score day3 |  |  |
| Respiratory | 0.966 (0.805-1.158) | 0.706 |
| Hematologic | 0.864 (0.647-1.153) | 0.321 |
| Hepatic | 0.827 (0.594-1.151) | 0.26 |
| Cardiovascular | 0.892 (0.784-1.014) | 0.08 |
| Neurologic | 1.102 (0.909-1.336) | 0.321 |
| Renal | 1.013 (0.821-1.249) | 0.906 |
| SOFA score day7 |  |  |
| Respiratory | 1.372 (1.154-1.631) | <0.001 |
| Hematologic | 1.573 (1.239-1.997) | <0.001 |
| Hepatic | 1.356 (1.044-1.762) | 0.022 |
| Cardiovascular | 1.331 (1.152-1.538) | <0.001 |
| Neurologic | 1.358 (1.131-1631) | 0.001 |
| Renal | 1.236 (1.029-1.485) | 0.023 |
| 181 patients were excluded from the analysis since SOFA score on day 3 and 7 was not recorded. Since the number of patients was small, intra-aortic balloon pumping was not included in the logistic regression model.  Abbreviation: DIC; disseminated intravascular coagulation, ECMO; extracorporeal membrane oxygenation, RBC; red blood cell concentration, FFP; fresh frozen plasma, CI; confidence Interval | | |
|  |  |  |
|  |  |  |
|  |  |  |
|  |  |  |
|  |  |  |
